# Involvement of P2X7 receptor signaling on regulating the differentiation of Th17 cells and type II collagen-induced arthritis in mice

**DOI:** 10.1038/srep35804

**Published:** 2016-10-24

**Authors:** Zhi-Dan Fan, Ya-Yuan Zhang, Yi-Hong Guo, Na Huang, Hui-Hui Ma, Hui Huang, Hai-Guo Yu

**Affiliations:** 1 Department of Rheumatology and Immunology, Children’s Hospital of Nanjing Medical University, No. 72 Guangzhou Road, Nanjing, Jiangsu Province 210008, China

## Abstract

Interleukin (IL)-17 producing T helper (Th17) cells are major effector cells in the pathogenesis of rheumatoid arthritis (RA). The P2X7 receptor (P2X7R) has emerged as a potential site in the regulation of inflammation in RA but little is known of its functional role on the differentiation of Th17 cells. This study investigates the *in vitro* and *in vivo* effects of P2X7R on Th17 cell differentiation during type II collagen (CII) induced experimental arthritis model. In CII-treated dendritic cells (DCs) and DC/CD4^+^ T coculture system, pretreatment with pharmacological antagonists of P2X7R (Suramin and A-438079) caused strong inhibition of production of Th17-promoting cytokines (IL-1β, TGF-β1, IL-23p19 and IL-6). Exposure to CII induced the elevation of mRNAs encoding retinoic acid receptor-related orphan receptor α and γt, which were abolished by pretreatment with P2X7R antagonists. Furthermore, blocking P2X7R signaling abolished the CII-mediated increase in IL-17A. Blockade of P2X7R remarkably inhibited hind paw swelling and ameliorated pathological changes in ankle joint of the collagen-induced arthritis mice. Thus, we demonstrated a novel function for P2X7R signaling in regulating CII-induced differentiation of Th17 cells. P2X7R signaling facilitates the development of the sophisticated network of DC-derived cytokines that favors a Th17 phenotype.

Rheumatoid arthritis (RA), characterized by joint inflammation and destruction, is a chronic and systemic autoimmune disorder of unknown etiology^1^. It affects approximately 1% of the population worldwide^2^. Patients developing RA typically present with morning stiffness, joint deformity, limited range of motion, weight loss, ligamentous laxity, fatigue, etc^3^,^4^. RA has been associated with reduced quality of life, and can influence the activity of other organ systems, including the cardiovascular, pulmonary and gastrointestinal systems^5^,^6^. Multiple new treatment options have been greatly developed in recent years due to many advances in the diagnosis, management and understanding of RA^7^,^8^. Even though these therapeutic measures have effectively attenuated the symptoms and modified the course of RA, a significant proportion of patients may still not reach the desired therapeutic target^9^. Therefore, the searching for additional therapeutic candidates using a unique mechanism remains the top priority for RA therapy.

Inflammatory T cells have been shown to play central roles in the pathogenesis of RA^10^,^11^. Accumulating evidence indicates that inflammatory T cells and their proinflammatory cytokines are present early in the disease process in the joint synovium^12^. These alterations contribute to pathogenesis in the progression of an early synovial inflammation towards a chronic arthritis^13^. Thus, inflammatory T cells, especially T helper (Th) cells, have gained wide interest as treatment targets^14^. Interleukin (IL)-17 producing Th (Th17) cells, potent inducer of tissue inflammation, are currently recognized as major effector cells in the pathogenesis of RA^15^,^16^. In addition, Th17 cells could stimulate the release of proinflammatory cytokines to promote inflammation via IL-17^17^. In RA, the expression of receptor activator nuclear kappa ligand was upregulated by Th17 cells, resulting in the bone erosion and consequent joint destruction^18^. We failed to induce the arthritis model in IL-17 receptor knockout mice in our preliminary study. Collectively, Th17 cells are involved in multiple pathological processes of RA, and regulation on Th17 cell differentiation may be a good therapeutic strategy in the treatment of RA.

It is now widely accepted that adenosine triphosphate (ATP) is an important extracellular signaling molecule, which has been implicated in a wide variety of biological processes via P2 receptors^19^,^20^. The P2X7 receptor (P2X7R) is an ATP-gated ion channel belonging to P2 receptors, and has emerged as a potential site in the regulation of inflammation in RA^21^. Particularly, the P2X7R allows cation passage through the cell to activate the inflammasomes and production of IL-1β, which is obligatory for the Th17 development^22^,^23^. These findings provide the functional basis supporting the hypothesis that P2X7R may regulate the *in vitro* and *in vivo* effects of type II collagen (CII) on the differentiation of Th17 cells.

To clarify the role of P2X7R in regulating CII-induced differentiation of Th17 cells, we assessed the contribution of P2X7R signaling on cytokine production during initial T cell priming in CII-treated dendritic cells (DCs). In addition, the effect of P2X7R signaling on the Th17 cell differentiation was investigated on the CD4^+^ T cells in coculture with DCs. The animal study was also performed to assess the effectiveness of blocking P2X7R signaling to suppress Th17 cell differentiation.

## Methods

### Human subjects

A total of 19 patients with juvenile RA (8 males, 11 females; mean age 8.71 ± 3.26 years), and 22 healthy age- and sex-matched volunteers served as controls (10 males, 12 females; mean age 8.92 ± 3.45 years) were enrolled into the study at the Nanjing Children’s Hospital Affiliated to Nanjing Medical University. All patients and volunteers were Han Chinese. All patients were clinically assessed according to the detailed diagnostic information obtained from the medical records and physical examinations. The study was approved by the Ethics Committee of Nanjing Medical University (Nanjing, China) and all methods were performed in accordance with the relevant guidelines and regulations. Written informed consent on the use of clinical specimens for medical research was obtained from the parents or legal guardians of the participants. The peripheral blood mononuclear cells (PBMCs) from all study participants were isolated and cultured for the measurement of the P2X7R expression.

### Experimental animals

Male DBA/1J mice were purchased from the Shanghai SLAC Laboratory Animal Co., Ltd. (Shanghai, China). All mice were kept under a light-, temperature- and humidity-controlled environment with a constant 12-h light/dark cycle, and access to food and water ad libitum. All animal procedures and study protocols were approved by the Experimental Animal Care and Use Committee of Nanjing Medical University and complied with the Guide for the Care and Use of Laboratory Animals (NIH Publication No. 85-23, revised 1996).

### Generation of DCs

Bone marrow-derived DCs were prepared as described previously^24^. Briefly, bone marrow precursor cells were obtained from femur and tibia of mice and cultured in fresh DC culture medium consisting of RPMI 1640 medium plus granulocyte-macrophage colony-stimulating factor (10 ng/ml; Immunex, Seattle, WA, USA) and IL-4 (1 ng/ml; PeproTech, Rocky Hill, NJ, USA). DCs were induced to mature by lipopolysaccharide (100 ng/ml; Sigma-Aldrich, St Louis, MO, USA).

### Measurement of extracellular ATP

The concentration of extracellular ATP was detected using ENLITEN^®^ rLuciferase/Luciferin Reagent (Promega, Madison, WI, USA). DCs (2.5 × 10^5^ cells/well) were incubated in RPMI 1640 medium supplemented with 1% (v/v) fetal bovine serum for 12 h in a 12-well culture plate. An aliquot of 40 μl conditioned medium was collected as a control sample for baseline ATP release. The cells were exposed to bovine CII (Chondrex, Redmond, WA, USA) at room temperature, and then conditioned medium was collected at the indicated time points. Each sample was centrifuged at 600 g for 5 min at 4 °C and 10 μl of the supernatant was used for ATP determination. The concentration of ATP was determined by measuring the chemiluminescense with a SpectraMax luminometer (Molecular Devices, Downingtown, PA, USA) according to manufacturer’s instructions.

### Quantitative real-time PCR

RNA extraction and quantitative real-time PCR were performed for gene expression analysis as previously reported^25^. Briefly, total RNA was extracted from cell samples with TRIzol reagent (Invitrogen, Carlsbad, CA, USA) in accordance with the manufacturer’s protocols. The concentration of RNA samples was determined in duplicate by measuring the optical density (OD) at 260 nm. Purity of RNA was ensured by an OD260/OD280 ratio in the range of 1.8–2.1. RNA was reversely transcribed to synthesize cDNA using the cDNA synthesis kit (Fermentas, St. Leon-Rot, Germany). One microliter of cDNA was then amplified in a 20-μl reaction mixture. The reaction was performed according to a modified protocol on the 7500 Fast Real-Time PCR System (Applied Biosystems, Foster City, CA, USA). The β-actin was adopted as an endogenous reference, and the 2^−ΔΔct^ method was used to calculate the relative expression level of interested genes in each group. Primer sequences were shown in Supplementary Table 1.

### Western blot analysis

Proteins were extracted and subjected to Western blot analysis as previously described^26^,^27^,^28^. Equal amounts of proteins (30 μg) were separated by a 10% SDS-PAGE and transferred to nitrocellulose membrane (Pall, Pensacola, FL, USA) by semi-dry blotting. Membranes were probed with the primary antibody against P2X7R (Santa Cruz Biotechnology, CA, USA) and visualized using an enhanced chemiluminescence procedure (Pierce, Rockford, Illinois, USA) with BioMax films (Kodak). The OD of each interested band was normalized to that of β-actin band by the Image-Pro^®^ Plus 4.5 software.

### Purification of CD4^+^ T cells

Single-cell suspensions from fresh spleen were harvested and prepared. Magnetic bead separation was used to negatively isolate CD4^+^ T cells. Briefly, single-cell suspensions were depleted by biotin-conjugated specific monoclonal antibodies against CD8^+^, B220^+^, CD16^+^, Gr-1^+^ and Ly76^+^ cells (BD Biosciences PharMingen, San Diego, CA, USA), anti-biotin magnetic beads, and an LD magnetic bead column (Miltenyi Biotec, Auburn, CA, USA)^24^. The purity of CD4^+^ T cells was consistently greater than 95% as assessed by flow cytometry.

### Co-culturing CD4^+^ T cell with DCs

DCs (2 × 10^4^ cells per well) incubated with and without two P2X7R antagonists (Suramin and A-438079) were cultured with CD4^+^ T cells (1 × 10^5^ cells per well) in the presence of CII (20 μg/ml) for 72 h. The supernatant was collected for cytokine measurement.

### Induction of CIA

CIA was induced in 6- to 8-week-old male DBA/1J mice as previously described^29^,^30^. Briefly, the mice were immunized intradermally at the tail base with bovine CII (100 μl of 1 mg/ml) emulsified in complete Freund’s adjuvant containing killed Mycobacterium tuberculosis (Difco, USA) (day 0). On day 21, animals were boosted with 200 μg of CII emulsified with incomplete Freund’s adjuvant at the base of the tail. From the primary immunization day, mice were treated with P2X7R antagonists (Suramin [30 mg/kg] and A-438079 [5 mg/kg]) or saline i.p every week for five weeks.

### Quantification of cytokines

Cell-free culture supernatant from coculture system was collected and the concentrations of IL-1β, transforming growth factor (TGF)-β1, IL-23p19, IL-12p40, IL-12p35 and IL-6 were measured by using specific ELISAs according to the protocol recommended by the manufacturer. A standard curve was generated for each plate over the linear range and used to calculate the absolute concentrations of the indicated cytokines.

### Flow cytometry analysis

To detect the level of IL-17A^+^ CD4^+^ T cells in the DC-T cell coculture system^31^, cells were incubated with 50 ng/ml phorbol myristate acetate (Sigma-Aldrich, St Louis, MO, USA) and 1 μM ionomycin (Sigma-Aldrich, St Louis, MO, USA) in the presence of 10 μg/ml brefeldin A (eBioscience, SanDiego, CA, USA) for 4 h at 37 °C. Then the cells were stained with anti-CD4-FITC (eBioscience, SanDiego, CA, USA) for 30 min at 4 °C. After incubation with the fixation/permeabilization solution, cells were stained with anti-IL-17A-APC (eBioscience, SanDiego, CA, USA) for 30 min and analyzed using a FACSCalibur flow cytometer (BD Biosciences) and the CellQuest software.

### Tissue preparation and histological examination

At the peak of CIA (about 35 days after first immunization), mice were sacrificed and their joints were processed for histopathological analysis as previously described^32^. The joints were histologically analyzed and scored, including (i) synovial hyperplasia, (ii) bone or cartilage destruction, (iii) inflammatory cells infiltrate and (iv) vascular proliferation^33^. The pathological changes were graded from 0 to 3 based on the severity of each index as follows: grade 0, no detectable changes; grade 1, mild changes; grade 2, moderate changes; grade 3, severe changes. The total histological score was the sum of the four items and ranged from 0 to 12.

### Statistical analysis

The SPSS 18.0 for Windows was employed in the data analysis. Excel (version 2007, Microsoft) was also adopted for preliminary data analysis. All data were expressed as mean ± SE of individual values. Comparisons between two observations in the same experimental protocol were assessed by Student paired *t* test. ANOVA followed by post hoc Bonferroni test was applied when multiple comparisons between different groups were made. A value of *P *< 0.05 was considered statistically significant.

## Results

### Increased expression of P2X7R in PBMCs from patients with RA

**Figure 1:**
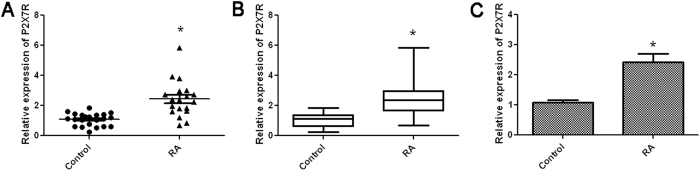
P2X7R had higher expression levels in the RA group than in the control group. The expression of P2X7R on PBMCs was measured by quantitative real-time PCR. (**A**) Scatter plot of relative expression of P2X7R. Each data-point represents the result for one normal individual or RA patient. Horizontal lines represent the mean value and bars represent the standard error; (**B**) Box-and-whisker plot with median value, interquartile range, and lower and upper values; (**C**) The graph shows the relative expression of P2X7R using the 2^−ΔΔCt^ method, with β-actin as the internal control. Data are presented as the mean ± SE from three independent experiments. *P < 0.05 *vs.* Control group.

We tested the expression of P2X7R on PBMCs in 19 patients with RA and 22 age-matched normal individuals using quantitative real-time PCR. As demonstrated in Fig. 1, P2X7R had higher expression levels in the RA group than in the control group, indicating that the P2X7R expression specifically unregulated in RA patients.

### Involvement of P2X7R in CII-induced ATP release from DCs

**Figure 2:**
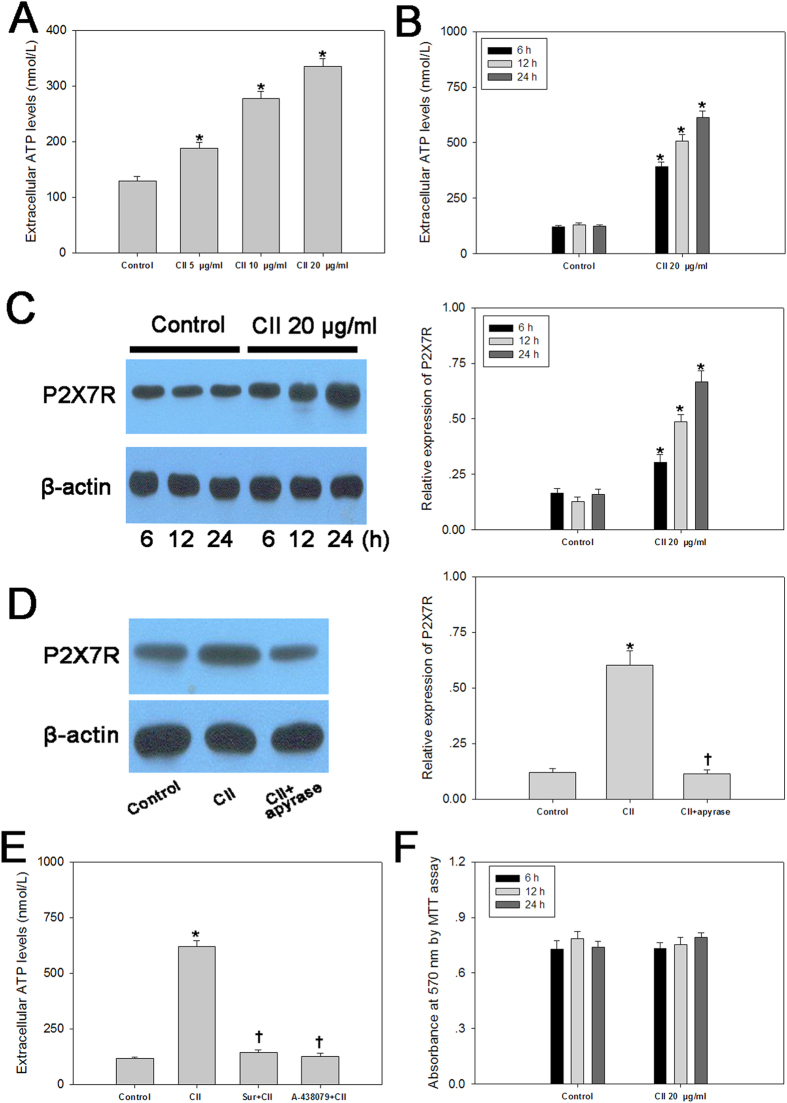
P2X7R was involved in CII-induced ATP release from DCs. (**A**) Changes of extracellular ATP concentrations in response to 5-minute treatment of CII (5, 10, 20 μg/ml); Extracellular ATP concentrations (**B**) and expression of P2X7R (**C**) in DCs cultured with 20 μg/ml CII for 6, 12 or 24 h; (**D**) Expression of P2X7R in CII-stimulated DCs with or without pretreatment by apyrase; (**E**) Extracellular ATP levels in CII-stimulated DCs with or without pretreatment by P2X7R antagonists; (**F**) Cell viability in DCs cultured with 20 μg/ml CII for 6, 12 or 24 h. Data are expressed as mean ± SE. Experiments were performed in triplicate and repeated twice. *P < 0.05 *vs.* Control group, ^†^*P *< 0.05 *vs.* CII group.

To investigate whether CII can stimulate the ATP release from DCs, the extracellular ATP levels of cells exposed to CII were investigated. In response to 5-minute treatment of CII, the extracellular ATP concentrations were increased in a dose-dependent manner as compared with unstimulated controls (Fig. 2A). Extracellular ATP levels were also measured in DCs cultured with 20 μg/ml CII for 6, 12 or 24 h. In control wells, ATP levels remained constant, whereas continuous treatment increased ATP levels up to 6 folds (Fig. 2B). Furthermore, CII treatment groups exhibited significantly higher P2X7R levels than the control group (Fig. 2C). In DC cultures treated with apyrase (an ATP diphosphohydrolase, 0.5 U/ml), CII failed to elevate the level of P2X7R, ruling out the possible involvement of endogenous ATP (Fig. 2D). Pretreatment with P2X7R antagonists (Suramin and A-438079) abolished the promoting effect of CII on ATP release from DCs (Fig. 2E). In the early stages of culture, cell viability was unaffected by CII treatment (Fig. 2F). So, the results reveal that CII-induced ATP release from DCs require the presence of P2X7R function.

### Involvement of P2X7R in CII-induced ATP release from DC/CD4^+^ T cell coculture system

**Figure 3:**
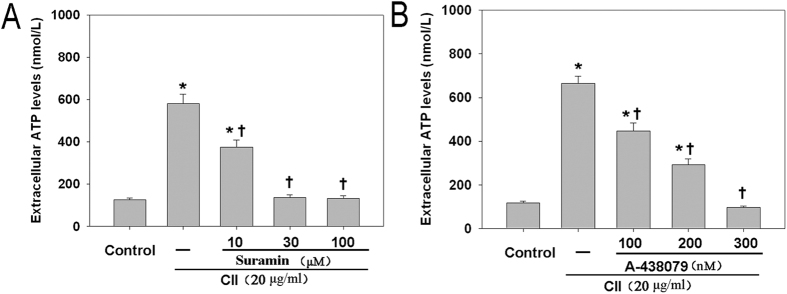
P2X7R was involved in CII-induced ATP release from DC/CD4^+^ T cell coculture system. Extracellular ATP levels in coculture system with or without pretreatment by (**A**) Suramin (10, 30 and 100 μM) or (**B**) A-438079 (100, 200 and 300 nM). Data are expressed as mean ± SE. Experiments were performed in triplicate and repeated twice. *P < 0.05 *vs.* Control group, ^†^*P *< 0.05 *vs.* CII group.

We preliminarily studied the effects of three doses (10, 30 and 100 μM) of Suramin and three doses (100, 200 and 300 nM) of A-438079 on the extracellular ATP levels in coculture system. Suramin completely blocked the CII-stimulated ATP secretion at the doses of 30 and 100 μM (Fig. 3A). We chose a lower dose of 30 μM of Suramin in this study to avoid potential toxicity. Only A-438079 at 300 nM could completely block the CII-stimulated P2X7R activation (Fig. 3B). Thus, 30 μM Suramin and 300 nM A-438079 were administered in the following experiments to ensure a better competitiveness. These results show that P2X7R is involved in the CII-induced ATP release from DC/CD4^+^ T cell coculture system.

### Increased production of Th17-promoting cytokines in the presence of CII

**Figure 4:**
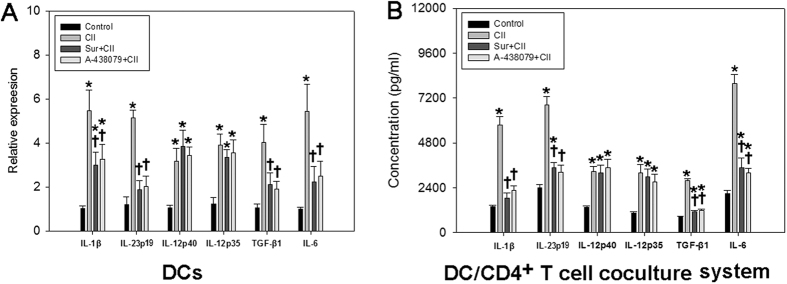
P2X7R was required for the induction of a Th17-promoting microenvironment by CII. (**A**) mRNA levels of IL-1β, TGF-β1, IL-23p19, IL-12p40, IL-12p35 and IL-6 in CII-stimulated DCs with or without pretreatment by P2X7R antagonists; (**B**) Supernatant levels of IL-1β, TGF-β1, IL-23p19, IL-12p40, IL-12p35 and IL-6 in CII-stimulated DC/CD4^+^ T cell coculture system with or without pretreatment by P2X7R antagonists. Data are expressed as mean ± SE. Experiments were performed in triplicate and repeated twice. *P < 0.05 *vs.* Control group, ^†^*P *< 0.05 *vs.* CII group.

To assess the role of CII and P2X7R signaling on cytokine production during Th17 cell differentiation, the marrow-derived DCs were cultured in the presence of CII with or without P2X7R antagonists. In response to CII treatment, we found substantial upregulation of the mRNAs encoding IL-1β, TGF-β1, IL-23p19, IL-12p40, IL-12p35 and IL-6 by approximately 2.5-6-fold above the unstimulated controls (Fig. 4A). We then blocked P2X7R signaling in DCs using P2X7R antagonists (Suramin and A-438079). Pretreatment with P2X7R antagonists caused strong inhibition of production of Th17-promoting cytokines (IL-1β, TGF-β1, IL-23p19 and IL-6), but not of Th1-promoting cytokines (IL-12p40 and IL-12p35) in CII-treated DCs, confirming the requirement of P2X7R for CII-induced development of a Th17-promoting rather than a Th1-promoting microenvironment.

In addition, we determined the ability of CII to induce cytokine production during Th17 cell differentiation in a DC/CD4^+^ T cell coculture system. Cytokine profile in the CII-stimulated coculture supernatant displayed a marked alteration compared with that of controls. We found strong production of the IL-1β, TGF-β1, IL-23p19, IL-12p40, IL-12p35 and IL-6, completely consistent with the changing trend of their mRNAs (Fig. 4B). Preconditioning with P2X7R antagonists only eliminated the release-promoting effects of CII on the above Th17-promoting cytokines (IL-1β, TGF-β1, IL-23p19 and IL-6), but had no effect on the production of IL-12p40 and IL-12p35. Thus, these data provide further evidence supporting the requirement of P2X7R for the induction of a Th17-promoting microenvironment by CII.

### Th17 cell differentiation induced by CII required P2X7R signaling

**Figure 5:**
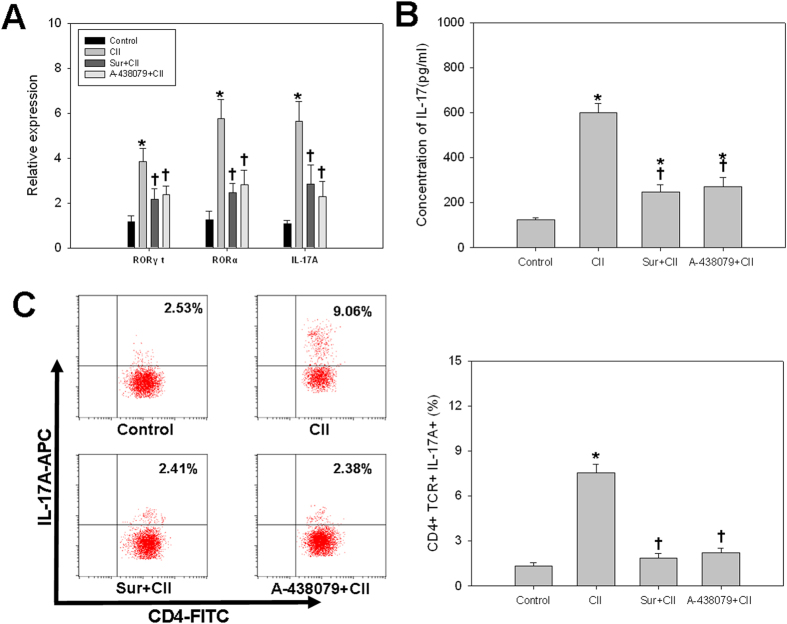
Th17 cell differentiation induced by CII required P2X7R signaling in DC/CD4^+^ T cell coculture system. (**A**) mRNA levels of RORγt, RORα and IL-17A; (**B**) Supernatant level of IL-17A; (**C**) Percentage of CD4^+^ IL-17A^+^ cells. Data are expressed as mean ± SE. Experiments were performed in triplicate and repeated twice. *P < 0.05 *vs.* Control group, ^†^*P *< 0.05 *vs.* CII group.

As the addition of CII could stimulate the production of Th17-promoting cytokines, we then evaluated the influence of CII on the induction of transcription factors essential for Th17 cell differentiation such as retinoic acid receptor-related orphan receptors α and γ (RORα and RORγt). Exposure to CII induced the elevation of mRNAs encoding RORγt and RORα whereas pretreatment with P2X7R antagonists abolished these increases (Fig. 5A). Further, CII treatment resulted in a substantial up-regulation of mRNA (Fig. 5A) and protein expression (Fig. 5B) of IL-17A, which was abrogated by antagonists of P2X7R. Moreover, in DC/CD4^+^ T coculture system, we found that CD4^+^ IL-17A^+^ cells were significantly increased in response to CII, whereas prior exposure to P2X7R antagonists had no effect on the CD4^+^ IL-17A^+^ cell populations in CII-stimulated subjects as compared with unstimulated controls (Fig. 5C).

**Figure 6:**
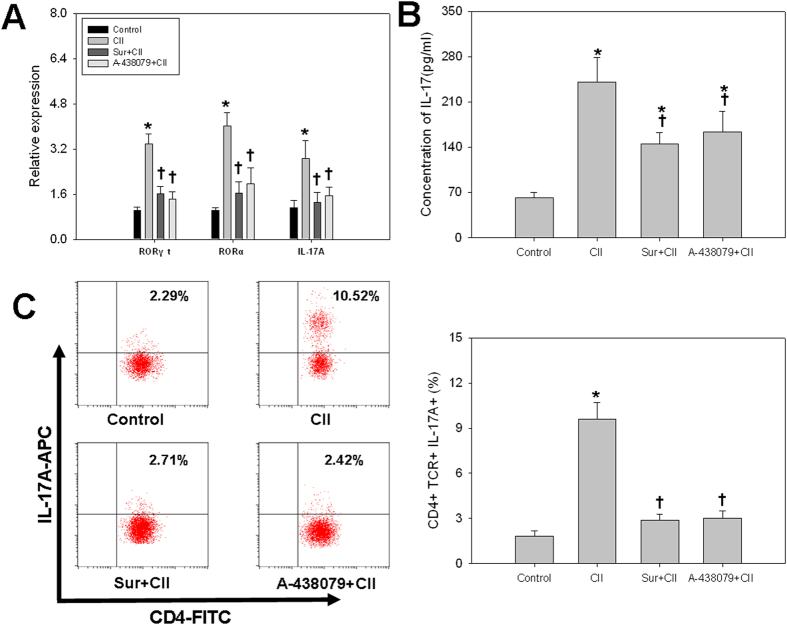
Th17 cell differentiation induced by CII required P2X7R signaling in CIA mice. (**A**) mRNA levels of RORγt, RORα and IL-17A in splenocytes; (**B**) Supernatant level of IL-17A in sera; (**C**) Percentage of CD4^+^ IL-17A^+^ cells in splenocytes. Data are expressed as mean ± SE. *n *= 8 for each group. *P < 0.05 *vs.* Control group, ^†^*P *< 0.05 *vs.* CII group.

Similarly, the mRNA (Fig. 6A) and protein expression (Fig. 6B) of IL-17A were increased remarkably in splenocytes derived from CII-treated mice. Consistently, the expression of Th17-transcriptional factors RORα and RORγt were increased in CII-treated mice (Fig. 6A). Furthermore, CII treatment resulted in a significantly higher percentage of CD4^+^ IL-17A^+^ cells (Fig. 6C). These data signified that CII promoted the Th17 cell differentiation in mice. However, pretreatment with P2X7R antagonists could abolish CII-induced Th17 cell differentiation in mice.

**Figure 7:**
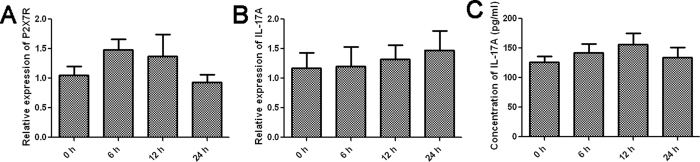
IL-17 elevation was not caused by the direct effect of ATP on T cells. (**A**) Changes of the P2X7R expression on T cells cultured with 20 μg/ml CII for 6, 12 or 24 h by quantitative real-time PCR; The mRNA (**B**) and protein (**C**) levels of IL-17A in response to 3 mM ATP after the induction of Th17 polarization of CD4^+^ T cell *in vitro*. Data are expressed as mean ± SE. Experiments were performed in triplicate and repeated twice.

To investigate the possibility that the IL-17 elevation is due to the direct effect of ATP on T cells, we induced Th17 polarization of CD4+ T cell *in vitro* and measured the mRNA and protein levels of IL-17A in response to 3 mM ATP. Besides, we measured the expression of P2X7R on T cells cultured with 20 μg/ml CII for 6, 12 or 24 h by quantitative real-time PCR. It was found that CII caused no change of P2X7R mRNA in T cells as compared with unstimulated controls (Fig. 7A). The extracellular ATP failed to induce IL-17A expression both at the transcriptional and translational levels (Fig. 7B,C), thus excluding the possibility that the IL-17 elevation is due to the direct effect of ATP on T cells. Combined with these findings, we believe that IL-17A elevation after CII treatment might be a consequence of the enhanced expression of Th17 related cytokines released by DCs.

### Blocking the P2X7R signaling attenuated CII-mediated joint damage of collagen-induced arthritis (CIA) mice

**Figure 8:**
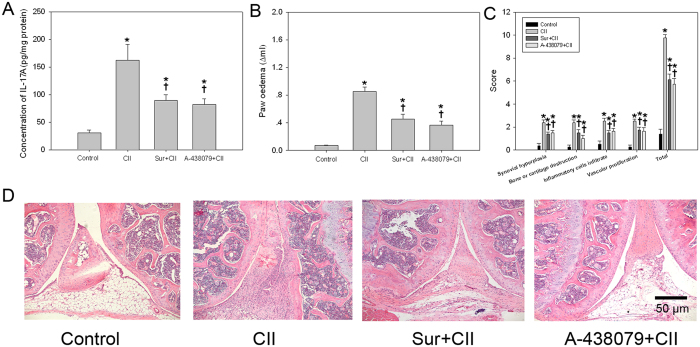
Blocking the P2X7R signaling attenuated CII-mediated joint damage of CIA mice. (**A**) Level of IL-17A in synovial tissues; (**B**) Paw oedema; (**C**) Histological analysis of joints; (**D**) histological images stained with H&E. Data are expressed as mean ± SE. *n *= 8 for each group. *P < 0.05 *vs.* Control group, ^†^*P *< 0.05 *vs.* CII group.

Compared with the control subjects, there was a higher level of IL-17A in synovial tissues in CII-treated mice (Fig. 8A). As shown in Fig. 8B, significant increases in paw oedema were recorded in CIA mice rather than controls. Histological score calculated from microphotographs was depicted in Fig. 8C. In addition, histological images suggested that CII leaded to severe synovial hyperplasia, bone or cartilage destruction, vascular proliferation and inflammatory cells infiltration (Fig. 8D). Treatment with P2X7R antagonists was effective in suppressing the severity of arthritis and immune responses to CII. These results suggested that blocking the P2X7R signaling attenuated joint damage of CIA mice *in vivo*.

## Discussion

The primary novel findings of the present study were that P2X7R was involved in CII-induced ATP release from DCs, and required for CII-induced development of a Th17-promoting microenvironment. Furthermore, we found that CII regulated the Th17 cell differentiation by mediating P2X7R signaling both *in vivo* and *in vitro*. Blocking the P2X7R signaling could attenuate CII-mediated joint damage of CIA mice. To our knowledge, this is the first study supporting the requirement of P2X7R for the regulation of Th17 cell differentiation by CII.

As an important extracellular signaling molecule, ATP is a potent danger-associated molecular pattern molecule that may contribute to tissue damage and inflammation^19^,^20^,^34^. Extracellular ATP signaling acting through the P2X7R played an important regulatory/modulatory role in the pathogenesis of RA^35^. In RA patients, a high concentration of extracellular ATP is present in the synovial fluid^36^. Expression of P2X7R was higher in monocytes from patients with RA compared with controls^37^. Similarly, we also observed significantly increased P2X7R expression in the RA patients as compared to their normal counterparts. In addition, P2X7R deficiency leads to a lower incidence and lower severity of joint inflammation in animal models of arthritis^38^. Recently, it has become evident that P2X7R is involved in ATP release in many types of cells (astrocytes, osteoclasts, osteoblasts, etc.)^39^,^40^, and the release of ATP via the P2X7R is supposed to be an important source of extracellular adenosine^41^. In osteoclasts under normal conditions, efflux via the P2X7R is the primary mechanism for ATP releases^40^. Although exactly how up regulation of the P2X7R mediates ATP release from cells is unclear, a likely mechanism for ATP release is directly through the formation of membrane pores caused by transient activation of the P2X7R.

Nowadays, significant roles for P2X7R signaling in the therapies of RA are emerging. CIA, induced by immunization with an emulsion of complete Freund’s adjuvant and CII, is the most commonly studied autoimmune model of RA to identify potential pathogenic mechanisms of autoimmunity^42^. CIA resembles many pathological features of RA, including synovial hyperplasia, mononuclear cell infiltration and cartilage degradation. Of the antigen-defined models that are based on cartilage proteins, CIA has the shortest duration between immunization and disease manifestation^42^. CII is a major protein in cartilage, the target tissue of RA^42^. The presence of T-cell and B-cell immunity to CII has been well established in the immunopathogenesis of RA^43^,^44^,^45^. Here, we firstly investigated the role of CII on the ATP release from DCs. CII dose-dependently increased the concentrations of extracellular ATP and prolonged culture with CII resulted in a further elevation of extracellular ATP. Inflammasome/IL-1β dependent and independent pathways maybe two downstream cellular signaling mechanisms proposed for P2X7R stimulation^46^,^47^.

Interestingly, we also observed that the strong promoting effect of CII on the release of ATP was accompanied by a large increase in protein levels of P2X7R. Knowing that involvement of the P2X7R in ATP release has recently been reported in many cell types^48^, we tested the hypothesis that P2X7R function was required for the CII-induced ATP release from DCs. Our data showing that the P2X7R antagonists completely abolished CII-stimulated ATP release suggest that P2X7R mediates ATP release from DCs in response to CII. The effect of apyrase treatment on P2X7R expression strongly suggests a reciprocal relationship between the release of ATP and P2X7R function in response of DCs to CII. It has been reported that sustained stimulation of the P2X7R caused phenotypic changes, membrane permeabilization, and eventually cell death^49^, but we did not observe any significant cytotoxicity up to 24 h of exposure to CII. In particular, P2X7R activation also contributes to the Th17 cell differentiation. Based on these findings, we postulate that P2X7R is involved in the CII-mediated Th17 polarization.

The cytokines IL-1β, TGF-β1, IL-23 and IL-6 are sufficient to differentiate Th17 cells from naive CD4^+^ T cells^50^,^51^, while IL-12 is a key player in polarizing CD4^+^ T cells toward the Th1 subset^51^. P2X7R activation enhances the release of Th17-biasing cytokines^50^,^51^,^52^. Our data add to this observation by showing that stimulation of P2X7R by CII strongly potentiated the production of IL-1β, TGF-β1, IL-23 and IL-6 mRNA. In the absence of P2X7R signaling, stimulation with CII failed to induce the upregulation of Th17-promoting cytokines in DCs. A previous study showed that stimulation of P2X7R by BzATP caused no influence on the release of L-12p70^53^, and Miller *et al*.^54^ found that absence of P2X7R did not affect IL-12 production. In agreement with these data, we found that CII-stimulated DCs lacking functional P2X7R still possessed the ability to promote the production of IL-12p40 and IL-12p35. In contrast, absence of the P2X7R did not affect IL-1β production^54^, different from our findings in the current study. We speculated that different mediums measured in two studies may explain the discrepancy. Notably, the above results indicated that P2X7R was required for CII-induced development of a Th17-promoting rather than a Th1-promoting microenvironment. Interestingly, a study by Vergani *et al*.^50^ found that *in vitro* genetic upregulation of the P2X7R pathway was shown to stimulate Th1/Th17 cell generation. These findings suggest that there may be different roles of P2X7R for the differentiation of Th1 and Th17 cells under certain circumstances. Co-culturing system of CD4^+^ T cell and DCs is widely used to study the mechanisms underlying the polarization of naive CD4^+^ T cells *in vitro*^55^. We thus investigated the potential roles of P2X7R on the Th17 cell differentiation in this co-culturing system. Consistent with the mRNA findings, blocking the P2X7R signaling had a similar influence on the cytokine profile in the CII-stimulated coculture supernatant. The ELISA results enhanced our finding that P2X7R was necessary in the CII-mediated Th17 cell differentiation.

Extracellular ATP signaling acting through the P2X7R is a complex and dynamic scenario^56^. A previous study showed that ATP stimulation strongly induced P2X7R expression both at the transcriptional and translational levels^57^. Subsequently, pores formed in response to P2X7R activation induced additional ATP release, initiating a positive feedback loop^58^. From the results of *in vitro* experiments, we have found a reciprocal relationship between ATP release and P2X7R function in response to CII, by which CII-induced P2X7R activation promote the ATP release via the formation of membrane pores, meanwhile intensified stimulation of ATP enhance the expression of P2X7R. The dynamic association between ATP release and P2X7R expression strengthens their functions against challenge with CII. As previously reported, ATP activated the P2X7R to induce cytokine responses in inflammatory conditions^59^. In this work, we have explored the release of six inflammatory cytokines (IL-1β, TGF-β1, IL-23, IL-12p40, IL-12p35 and IL-6) from DCs in response to CII. Considerable research effort has focused on the possible mechanism downstream of ATP-P2X7R for the induction of inflammatory cytokines, including NLRP3 inflammasome-dependent IL-1β secretion^60^, PKC/MAPK signalling pathway for TGF-β1^61^ and p38 MAPK pathway for IL-6^62^. Collectively, we believe the activation of downstream signals of ATP-P2X7R axis is likely responsible for the release of inflammatory cytokines induced by CII. Further studies are needed to elucidate signalling pathways for the release of inflammatory cytokines induced by CII.

Given that P2X7R signaling facilitated the development of cytokine milieu important for Th17 cell differentiation, we then investigated its influence on the expressions of RORγt and RORα, essential for Th17 cell differentiation^55^. We firstly reported that the mRNAs levels of RORγt and RORα were profoundly elevated following the stimulation of P2X7R by CII. Furthermore, we failed to observe the upregulation of the mRNAs encoding RORγt and RORα in DC/CD4^+^ T coculture system after the blockade of P2X7R, hinting that P2X7R could positively regulate the expressions of RORγt and RORα.

Blocking P2X7R signaling abolished the CII-mediated increase in IL-17A. Importantly, no change was observed in response to CII in the proportion of CD4^+^ IL-17A^+^ cells by treatment with P2X7R antagonists compared with unstimulated controls, proving the sufficient and direct evidence that the P2X7R is a necessary component for the CII-induced differentiation of Th17 cells. Further analysis in spleen T cells from CIA mice showed the same change trends of the above indices for Th17 cell differentiation. Based on these findings, we propose that CII promotes the differentiation of Th17 cells via P2X7R signaling both *in vivo* and *in vitro*.

Interestingly, recent studies suggested the possible involvement of B cells in IL-17 production. Schlegel *et al*.^63^ demonstrated that in addition to Th17 cells, a substantial number of other innate and adaptive immune cells, in particular also B cells participate in the IL-17 production during established RA. Another study also identified B cells as a major source for rapid, innate-like IL-17 production *in vivo* in response to *Trypanosoma cruzi* infection^64^. P2X7R is expressed on cells of the immune system, including CD4 T cells and B cells^65^. However, it is still unknown whether P2X7R antagonist effect is only specific to CD4 T cells rather than B cells. We speculate that it is possible that P2X7R antagonists induced both inhibition of CD4 T cells and B cells may work simultaneously to abolish the CII-mediated increase in IL-17A *in vivo*.

Considering the positive effect of P2X7R signaling in the differentiation of Th17 cells, we next examined whether P2X7R inhibition attenuated CII-mediated joint damage of CIA mice. Our results indicated that blockade of P2X7R remarkably inhibited hind paw swelling and ameliorated the pathological changes in ankle joint of the CIA mice. These results clearly suggest that P2X7R might be an interesting target for the clinical treatment of RA. Since the blockade of P2X7R can attenuate the differentiation of Th17 cells promoted by CII *in vivo* and *in vitro*, further application of therapeutic regimen targeting P2X7R should be investigated.

Recently, it has been recognized that GM-CSF has the potential in the amelioration of autoimmune diseases by inducing natural Tregs. As previously reviewed, although blocking GM-CSF or its receptor has been regarded as available potent anti-inflammatory therapies, GM-CSF can act as an anti-inflammatory/regulatory cytokine in many situations^66^,^67^. It can increase regulatory T-cell numbers and function by modulating differentiation of DCs^68^. Interestingly, Gathungu *et al*.^69^ demonstrated that elevated GM-CSF autoantibody was a risk marker for aggressive Crohn’s disease behavior and complications including surgery, indicating that deficiency of GM-CSF can contribute to a relative immunodeficiency. A case report by Rowin *et al*.^70^ revealed that GM-CSF treatment leaded to a sustained increase in the Foxp3 expression and an enhance capability of Foxp3+CD4+CD25+ Tregs to limit the proliferative capacities of CD4+ T effector cells. Our study showed that P2X7R presented positive effect on the differentiation of Th17 cells through its pro-inflammatory role and P2X7R inhibition attenuated joint damage of CIA mice. Given the opposite effects of GM-CSF and P2X7R on the regulation of Tregs/Th17 balance, we postulate GM-CSF may possibly affect the expression of P2X7R and hence attenuate CIA. Further experiments are needed to test the hypothesis.

In addition to the induction of CIA, CII is proved to be capable of generating eye-mediated immune tolerance^71^ and ACAID-mediated immune tolerance^72^. The P2X7R is emerging as a novel anti-inflammatory therapeutic target, and various selective P2X7R antagonists are under clinical trials. In view of the potential role of P2X7R in the regulation of inflammation reported in our study and other previous studies^35^,^38^,^41^,^46^,^54^, we believe that P2X7R may have a central role in the above immune tolerance. This could have therapeutic implications in other routes of immune tolerance in which CII is involved.

In summary, we have demonstrated a novel function for P2X7R signaling in regulating CII-induced differentiation of Th17 cells. P2X7R signaling facilitates the development of the sophisticated network of DC-derived cytokines that favors a Th17 phenotype. As DC is of critical importance in immunotherapy, P2X7R may become an interesting target for DC-based therapeutic approaches. Fortunately, several potent P2X7R antagonists are available that could be helpful for drug screening and in managing patients with RA.

## Additional Information

**How to cite this article**: Fan, Z.-D. *et al*. Involvement of P2X7 receptor signaling on regulating the differentiation of Th17 cells and type II collagen-induced arthritis in mice. *Sci. Rep.* **6**, 35804; doi: 10.1038/srep35804 (2016).

## Supplementary Material

Supplementary Information
