# Supplementary material for: Involvement of P2X7 receptor signaling on regulating the differentiation of Th17 cells and type II collagen-induced arthritis in mice
Source: Sci Rep. 2016 Oct 24;6:35804. doi: 10.1038/srep35804 (PMC5075966; doi:10.1038/srep35804)
Supplement: Supplementary Information [file srep35804-s1.doc]

**Involvement of P2X7 receptor signaling on regulating the differentiation of Th17 cells and type II collagen-induced arthritis in mice**

Zhi-Dan Fan^1^, Ya-Yuan Zhang^1^, Yi-Hong Guo, Na Huang, Hui-Hui Ma, Hui Huang, Hai-Guo Yu*****

## Department of Rheumatology and Immunology, Nanjing Children’s Hospital Affiliated to Nanjing Medical University, No. 72 Guangzhou Road, Nanjing, Jiangsu Province 210008, China

## ^1^These authors contributed equally to this work

***Address for correspondence:**

Hai-Guo Yu, Ph.D., M.D., Chair

Department of Rheumatology and Immunology

Nanjing Children’s Hospital Affiliated to Nanjing Medical University, Nanjing 210008, China

Tel: +86-25-83116831

E-mail:yuhaiguo53@126.com

**Table 1 Specific primers used in real-time PCR analysis**

| ***Name*** | ***Primer*** ***^a^*** | Sequence(5’-3’) |
| --- | --- | --- |
| IL-1β | F | GCAACTGTTCCTGAACTCAACT |
|  | R | ATCTTTTGGGGTCCGTCAACT |
| TGF-β1 | F | AACAATTCCTGGCGTTACCTT |
|  | R | GAATCGAAAGCCCTGTATTCC |
| IL-23 | F | ATGCTGGATTGCAGAGCAGTA |
|  | R | ACGGGGCACATTATTTTTAGTCT |
| IL-12p40 | F | ACATCAAGAGCAGTAGCAGTTCC |
|  | R | CAGTTGGGCAGGTGACATCC |
| IL-12p35 | F | CTGGAACTACACAAGAACGAGAG |
|  | R | CTTCAAGTCCTCATAGATGCTACC |
| IL-6 | F | TAGTCCTTCCTACCCCAATTTCC |
|  | R | TTGGTCCTTAGCCACTCCTTC |
| IL-17A | F | CTCCAGAAGGCCCTCAGACTAC |
|  | R | AGCTTTCCCTCCGCATTGACACAG |
| RORα | F | TCTCCCTGCGCTCTCCGCAC |
|  | R | TCCACAGATCTTGCATGGA |
| RORγt | F | CACGGCCCTGGTTCTCAT |
|  | R | GCAGATGTTCCACTCTCCTCTTCT |
| P2X7R | F | AAGCTGTACCAGCGGAAAGA |
|  | R | GCTCTTGGCCTTCTGTTTTG |
| β-actin | F | TGTTACCAACTGGGACGACA |
|  | R | GGGGTGTTGAAGGTCTCAAA |

^a^ F, forward primer; R, reverse primer.
